# Potentials of saponins-based adjuvants for nasal vaccines

**DOI:** 10.3389/fimmu.2023.1153042

**Published:** 2023-03-20

**Authors:** Kai Chen, Ning Wang, Xiaomin Zhang, Meng Wang, Yanyu Liu, Yun Shi

**Affiliations:** ^1^ Department of Radiology and National Clinical Research Center for Geriatrics, West China Hospital, Sichuan University, Chengdu, Sichuan, China; ^2^ West China Biopharmaceutical Research Institute, West China Hospital, Sichuan University, Chengdu, Sichuan, China

**Keywords:** saponins, adjuvant, mucosal adjuvant, ISCOMs, adjuvant systems

## Abstract

Respiratory infections are a major public health concern caused by pathogens that colonize and invade the respiratory mucosal surface. Nasal vaccines have the advantage of providing protection at the primary site of pathogen infection, as they induce higher levels of mucosal secretory IgA antibodies and antigen-specific T and B cell responses. Adjuvants are crucial components of vaccine formulation that enhance the immunogenicity of the antigen to confer long-term and effective protection. Saponins, natural glycosides derived from plants, shown potential as vaccine adjuvants, as they can activate the mammalian immune system. Several licensed human vaccines containing saponins-based adjuvants administrated through intramuscular injection have demonstrated good efficacy and safety. Increasing evidence suggests that saponins can also be used as adjuvants for nasal vaccines, owing to their safety profile and potential to augment immune response. In this review, we will discuss the structure-activity-relationship of saponins, their important role in nasal vaccines, and future prospects for improving their efficacy and application in nasal vaccine for respiratory infection.

## 1 Introduction

The human respiratory tract is a common entry point for various infectious microorganisms, including viruses such as coronavirus, respiratory syncytial virus (RSV), and influenza virus, as well as bacteria such as *Streptococcus pneumoniae*, *Staphylococcus aureus*, *Acinetobacter baumannii*, and *Mycobacterium tuberculosis*, which cause a significant global health concern, especially for older, or immunocompromised people (1–3). Vaccination is a cost-effective and effective way to prevent these infections. Although systemic vaccines against severe acute respiratory syndrome coronavirus 2 (SARS-CoV-2) or influenza virus can induce robust systemic immunity, they are not sufficient to prevent virus transmission and only reduce the development of severe disease (4, 5). Thus, a vaccine regimen that promotes mucosal immune responses in respiratory tract is crucial in preventing pathogens invasion (5–7).

Nasal vaccines are considered as a promising strategy by inducing antigen-specific protective immune responses in both mucosal surfaces and throughout the body. The respiratory route has unique physiological and immunological characteristics, and the nasopharyngeal-associated lymphoid tissue (NALT) is a key induction site for mucosal immunity after nasal vaccination (8). Compared to common injectable vaccines, nasal vaccination is more likely to elicit a robust mucosal response, characterized by antigen-specific T cell response in combination with secretory immunoglobulin A (sIgA) (9). Secreted IgA helps bind and eliminate pathogens before they can cause an infection (7). In addition to inducing mucosal immune response, nasal vaccine can also elicit systemic immune responses (7). Therefore, nasal vaccines are an attractive strategy for combating pathogens that use the respiratory tract as an entry point into the body. Furthermore, nasal vaccination has several benefits, such as a larger mucosal surface area for antigen uptake and convenience of being needle-free and easy to self-administer.

So far, only three nasal vaccines have been licensed for human use. The first is FluMist™, an attenuated influenza virus vaccine, approved by U.S. Food and Drug Administration (FDA) (10). The other two are SARS-CoV-2 vaccines, one is iNCOVACC™, a chimpanzee adenovirus-vectored SARS-CoV-2 vaccine licensed for restricted use in emergencies in India (11, 12); Another is CA4-dNS1-nCoV-RBD, a nasal spray influenza virus vector vaccine approved in China for emergency use (13). Despite the potential benefits of these vaccines, there are safety concerns since they use live viruses. Recombinant protein-based subunit vaccines are a safer alternative, but their immunogenicity is often weak, making it challenging to stimulate mucosal immunity. Meanwhile, the unique physiological and immunologic properties of the respiratory mucosa, such as the mucus layer and cilia movement, can pose obstacles in the development of nasal vaccines, and pH and enzyme conditions can degrade antigens (14). To overcome these challenges, adjuvants play a crucial role in enhancing antigen immunogenicity and vaccine efficacy in the respiratory mucosa (15).

Adjuvants are important components of vaccine formulations together with antigens and function to enhance the immunogenicity of the co-inoculated antigens to confer long-term and effective protection against pathogens (16). They can be broadly categorized into immunostimulatory molecules and delivery systems that transport the vaccines to the immune system (16). While various mucosal adjuvants such as cholera toxin, *Escherichia coli* heat-labile toxin, polyethyleneimine, alum, chitosan, and others have been tested in experimental subunit vaccines for respiratory infections, none of them have been approved for use in nasal vaccines (17).

Saponins, which are extracted from plants, have generated great interest as vaccine adjuvants due to their multiple biological and immunomodulatory properties (18). Quil A and its purified fraction QS-21 are the most widely used saponins as adjuvants, owing to their exceptional ability to enhance antibody responses and activate T helper type 1 cells (Th1) and cytotoxic T lymphocyte (CTL) immune responses by activating dendritic cells (DCs) (19, 20). Additionally, QS-21-based adjuvants, including adjuvant systems (AS01, AS02, et al.) and immunostimulating complexes (ISCOMs) have been developed with improved adjuvant effects and lower toxicity (19). AS01 has been successfully used as an adjuvant in the licensed vaccines, including the herpes zoster vaccine (Shingrix^®^) and the malaria RTS,S/AS01vaccine (Mosquirix™), showing their good efficacy and safety (20, 21). Currently, licensed adjuvants containing saponins are administrated intramuscularly. Saponins have also been used as adjuvants in nasal vaccines in several experiments and they are believed to have great potential to elicit strong mucosal and systemic immune responses (22). In this review, we aim to explain the structure-activity relationship of saponins, the crucial role of saponin-derived adjuvants in nasal vaccines, and provide insights into the future research on saponins-based adjuvants.

## 2 Chemical structure and effect of saponins

Saponins are a type of naturally occurring glycosides that are found in many plants. They consist of a steroid or triterpene trunk linked to one or more carbohydrate chains (23, 24). The structure of saponins varies greatly, which contributes to the diverse biological activities that they possess, such as immunomodulatory, anti-tumor, anti-inflammatory, antiviral, antifungal, cholesterol-lowering, and others (25). The adjuvant activity of saponins is mainly attributed to their ability to activate the mammalian immune system (25). Quil A, a saponin mixture extracted from the bark of the South American tree *Quillaja saponaria* Molina, has been widely used as an adjuvant due to its good adjuvant activity (26, 27). The purified fraction of Quil A, known as QS-21, was later separated using reversed-phase high performance liquid chromatography (RP-HPLC) and showed better adjuvant activity with lower toxicity (28). QS-21 has since become a popular adjuvant in vaccine studies and has been included in some approved vaccines (29).

**Figure 1:**
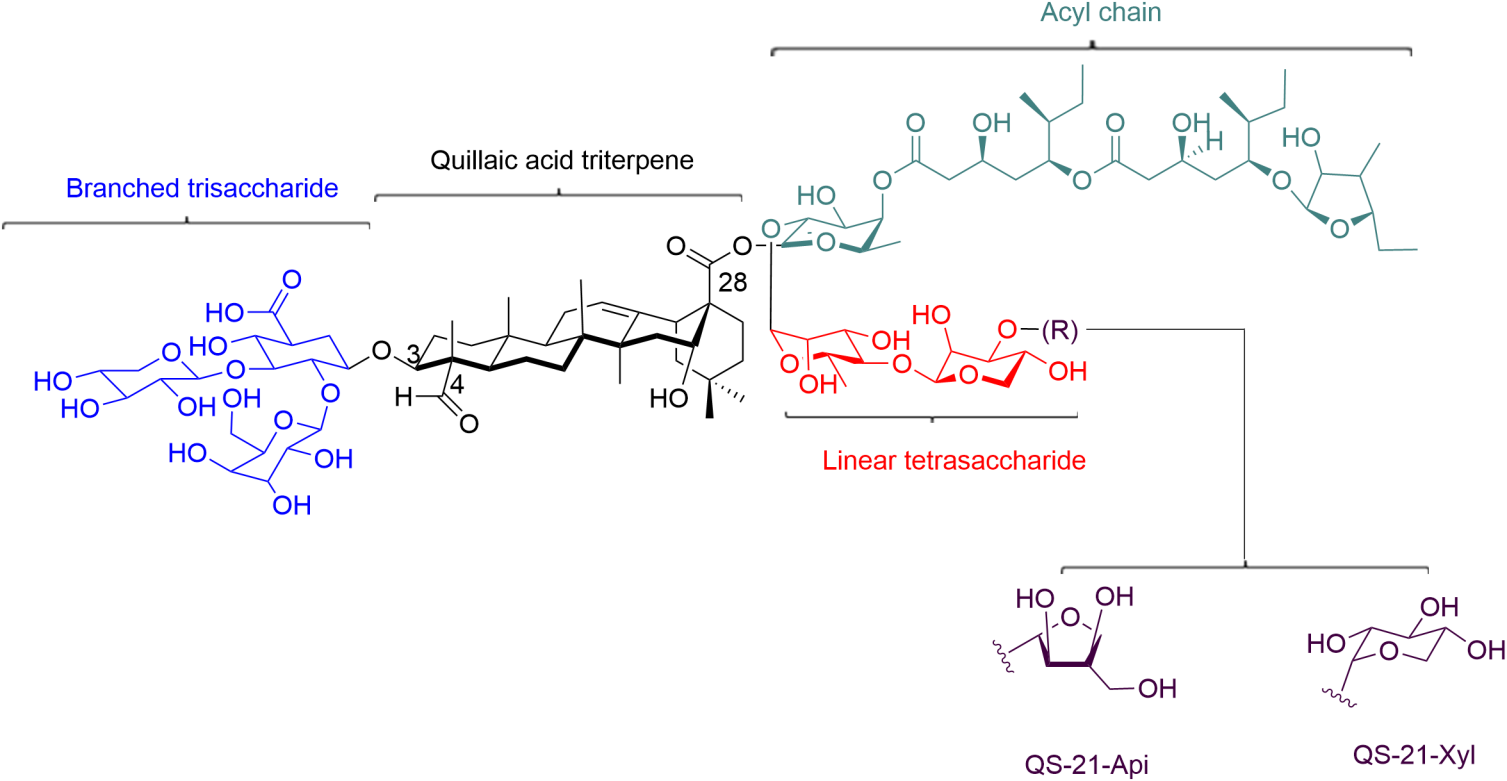
Structure of QS-21. QS-21 contains a central quillaic acid triterpene core, which is surrounded by complex oligosaccharide chains that are attached to C-3 and C-28 positions of the triterpene aglycone. The triterpene is essential for antigen cross-presentation and activation of innate immunity. The C4-aldehyde substituent on the triterpene is involved in the formation of Schiff base with amino groups on T cell surface receptors and providing a co-stimulatory signal to activate T cell. The acyl chain on QS-21 is associated with CTL responses. The branched trisaccharide is dispensable for activity. Linear tetracosaccharides can be structurally-modified to study the *in vivo* biological distribution of QS-21. Its carbohydrate domain involves in the uptake of antigen by APCs and the stimulation of specific cytokines that activate cellular and/or humoral responses.

QS21 is a triterpene glycoside that is soluble in water and consists of two isomers, QS-21 Apiose (QS-21 Api) and QS-21 Xylose (QS-21-Xyl) in a 2:1 ratio (**Figure 1**) (24). It features a central quillaic acid triterpene core, surrounded by complex oligosaccharide chains that are attached to C-3 and C-28 positions of the triterpene aglycone (**Figure 1**). The trisaccharide moiety at the C3 position is made up of d-glucuronic acid, d-galactose, and d-xylose. A linear tetrasaccharide, consisting of d-fucose, l-rhamnose, d-xylose, and either d-apiose or d-xylose (for QS-21Api or QS-21Xyl, respectively), is attached at the C-28 carboxylate of the triterpene *via* an ester bond. Finally, the triterpene saponins are completed by a structurally complex l-arabinose-terminated fatty acyl chain linked to the 4-position of the fucose residue, making them amphiphilic in nature (**Figure 1**) (30).

The underlying structure basis for the adjuvant activity of saponins has been extensively studied. Factors such as branched sugar chains, aldehyde groups, and an acyl residue on the aglycone can contribute to the adjuvant activity. QS-21 may bind to cell surface lectins through its carbohydrate domain, leading to the uptake of antigen by antigen-presenting cells (APCs) and the stimulation of specific cytokines that activate cellular and/or humoral responses (19, 31). The aldehyde group on the triterpene has been identified as crucial for adjuvanticity of saponins, as QS-21 derivatives modified at an aldehyde did not exhibit adjuvant activity for antibody stimulation or induction of CTL responses (32). The imine-forming carbonyl group can also form Schiff bases with amino groups on T cell surface receptors, leading to co-stimulation for T cell activation and inducing Th1 immunity and a CTL specific response (32, 33). The acyl chains on QS-21 have been associated with cytotoxic T-cell proliferation activity, since the removal of the acyl chain has been shown to be inactive for stimulation of antibody and CTL responses (24, 34). However, other saponins, such as soyasaponins and lablabosides, have been shown to have strong adjuvant activity despite lacking acyl residues (35). These studies suggest that acyl chain might contribute to the adjuvant activity, but not play an essential role. The amphiphilic structure of saponins, with a hydrophobic aglycone backbone and hydrophilic sugar side chains, has been related to the adjuvant activity (30, 36, 37). The triterpene’s affinity for cholesterol is essential for antigen cross-presentation and QS-21 destabilizes lysosomal membranes through cholesterol-dependent cytocytosis, leading to activation of innate immunity (19, 31, 38). Additionally, the amphiphilic nature of saponins makes them easy to formulation into other adjuvant complexes, such as liposomes or nanoparticle.

In addition to Quil A and QS-21, other plant sources of saponins with immune-stimulatory properties and low toxicities have been investigated, such as *Quillaja brasiliensis*, *Panax notoginseng*  *(* 39), *Panax ginseng* (40), *Platycodon grandiflorum* (41), *Pulsatilla chinensis* (42), *Soybeans* (43), *Polygala tenuifolia* (44), and *Paris polyphylla* (45). However, the limited availability of these sources, low isolation yields, and high purification costs have led to the exploration of other methods to obtain new saponins. Modification and synthesis of saponins are being developed to create more effective adjuvants with lower toxicity. The goal is to develop synthetic saponins with improved adjuvant-antigen activity and lower toxicity (46). For example, Shirahata et al. prepared a series of new simplified oleanolic acid saponins with a glycosyl ester moiety at C28 and found that cinnamoyl esterification of the glucose residue at C-28 was critical for providing mucosal adjuvant activity after intranasal immunization (46). Synthetic or semisynthetic saponins, such as GPI-0100, a semi-synthetic derivative of Quil A, and VSA-1, a newly developed semisynthetic analog of QS-21, have showed shows promising immunostimulatory activity in enhancing the immune responses (47–49).

## 3 The respiratory mucosal adjuvant effects of saponins

### 3.1 Adjuvant effects of plain saponins

Saponins are well-known for their ability to induce systemic immune responses when co-injected with antigen *via* intramuscular immunization (36). Additionally, saponins are effective as mucosal adjuvants when delivered intranasally (**Table 1**). Intranasal administration of a DNA vaccine encoding the envelope of human immunodeficiency virus type 1 (HIV-1) along with QS-21 as an adjuvant has been shown to increase both systemic and mucosal immune responses against HIV-1, including production of intestinal sIgA and cytolytic activity of mesenteric lymph node cells (50). Saponins derived from *Polygala tenuifolia* or *Chenopodium quinoa* have demonstrated enhanced antigen-specific immunoglobulin G (IgG) and local IgA responses to co-administered antigens in lungs when used as adjuvants following intranasal administration (44, 51). In another study, QS-21 loaded on liposome induced higher levels of sIgA compared to liposome without QS-21 when used as an adjuvant for a tetanus toxoid antigen after nasal administration (52). The use of oleanolic acid 28-cinnamoylglucoside as an adjuvant in a nasal-administered influenza split vaccine showed a slight but statistically significantly enhancement in anti-influenza virus sIgA in the nasal washes (46). Moreover, intranasal immunization of saponins-adjuvant vaccines showed protective effects against influenza virus and *Toxoplasma gondii* cysts challenge (44, 49, 53, 54). These studies highlight the potential of saponins as adjuvants for nasal vaccines.

**Table 1:** Plain saponins used as adjuvants in nasal vaccines.

Vaccine	Antigen	Adjuvant	Animal model	Pathogen Challenge	Main findings	Reference
**HIV-1 DNA vaccine**	Plasmid encoding the *env* and *rev* genes	QS-21	BALB/c mice	–	QS-21 can enhance the antigen-specific sIgA and promote Th1, CTL responses	(50)
**Model antigens**	Cholera toxin ovalbumin	Chenopodium quinoa saponins	BALB/c mice	–	Chenopodium quinoa saponins enhance the antibody responses to the co-administered proteins, possibly by increasing mucosal permeability	(51)
**Tetanus toxoid vaccine**	Tetanus toxoid	QS liposome	Rabbits	–	Tetanus toxoid plus QS liposomes induce higher sIgA levels in comparison with TT liposomes	(52)
**Influenza vaccine**	Influenza split vaccine	Cinnamoyl saponin 2	BALB/c mice	–	The synthetic saponins with the C28 4-O-cinnamoyl glucosyl ester moiety are efficacious vaccine adjuvants	(46)
**DPT or** **Influenza vaccine**	DPT HA	Onjisaponins	BALB/c mice	Influenza virus A/PR8 (H1N1)	Onjisaponins-adjuvanted vaccines induces serum IgG and nasal IgA antibody; Onjisaponins adjuvanted HA vaccine inhibites proliferation of influenza virus	(44)
***T.gondii* vaccine**	Crude rhoptry proteins	Quil-A	Cats	Cysts of the ME49 strain.	Quil-A–djuvanted vaccines yields more intestinal IgA antibodies and partially protect cats against *T. gondii* cysts challenge	(53)
***T.gondii* vaccine**	Crude rhoptry proteins	Quil-A	Pigs	Oocysts of VEG strain	Quil-A-adjuvanted vaccine induces serum IgG, IgM and stimulate a strong response in mesenteric lymph nodes, and partially protect animals from brain cyst formation	(54)

HIV-1, human immunodeficiency virus type 1; DPT, diphtheri–pertussis–tetanus; HA, Hemagglutinin; *T. gondii: Toxoplasma gondii*. "–" means "not done".

### 3.2 ISCOMs and ISCOMsATRIX (IMX)

ISCOMs was first described in the 1980s by Morein et al. as a novel type of immunostimulating complex. It has a spherical cage-like structure composed of saponin, cholesterol, phospholipids, and antigens (55, 56). On the other hand, ISCOMsATRIX (IMX), also called empty ISCOMs, has similar structure and composition to ISCOMs but without the incorporated antigens (56, 57). Both ISCOMs and IMX have been found to be highly immunostimulating due to the combination of an in-built adjuvant (Quil A) with a particulate delivery system. Moreover, they are less toxic and do not have hemolytic activity due to the embedding of saponins into cholesterol (56). The assembly of ISCOMs relies on hydrophobic interactions, making them suitable for incorporation of hydrophobic antigens derived from envelope viruses or cell membranes. In contrast, IMX has a negatively charged surface, which enables it easily to interact with a broad range of positively charged antigens to make IMX vaccines (57). However, this approach limits the binding of neutral or negatively charged hydrophilic antigens to IMX (57). Thus, further research is needed to expand the range of antigens available for IMX vaccines and simplify the production process (58). ISCOMs and IMX vaccines have been developed for a variety of diseases, including viruses, bacteria, parasites, and tumors (56). Both have shown good safety and well-tolerated in animal and human studies, with the ability to induce strong antigen-specific cellular or humoral immune responses (56, 59). There are several ISCOMs vaccines registered for veterinary use, but no ISCOMs or IMX vaccines have been approved for human use yet (60).

Studies have demonstrated that intranasal administration of ISCOMs or IMX vaccines with a variety of antigens can elicit potent mucosal cellular and humoral immune responses, including local IgG and sIgA antibodies in the respiratory tract, as well as systemic and distal mucosal responses, such as in the genital and intestinal tracts (61–70). Pulmonary delivery of ISCOMs or IMX vaccines has also been shown to induce both systemic and mucosal antibody responses against various antigens, including those of influenza virus and *Mycobacterium tuberculosis* (71–73). Protective efficacy of intranasal immunization with ISCOMs or IMX vaccines has been demonstrated in several studies against various pathogens such as influenza virus, *Helicobacter pylori*, *Angiostrongylus costaricensis*, and *Eimeria tenella* (74–80). These findings highlight the great potential of ISCOMs or IMX as adjuvants for nasal vaccine development (**Table 2**) (22).

**Table 2:** ISCOMs used as adjuvants in nasal vaccines.

	Vaccine	Antigen	Adjuvant	Animal model	Pathogen Challenge	Main findings	Reference
ISCOMs	Influenza vaccine	Glycoproteins	ISCOMs	Mice	–	ISCOMs vaccine induces similar serum IgG response and slightly higher IgA and IgM titres than that induced subcutaneously	(61)
	Influenza vaccine	Solubilized enveloped proteins	ISCOMs	BALB/c mice	–	ISCOMs vaccine induces influenza -specific antibody-secreting cells (ASC), memory B cell, and cytotoxic T cell responses in lung.	(62)
	HSV vaccine	gD2	ISCOMs IMX	BALB/c mice	–	gD2-ISCOMs induces mucosal antibody responses, even in the lower genital tract	(63)
	RSV vaccine	Solubilized virus	ISCOMs	BALB/c mice	–	RSV-ISCOMs induces high levels and long-lasting of IgA antibodies in respiratory tracts and also induce earlier, higher, and longer-lasting IgM and IgG1	(64)
	RSV vaccine	Envelope proteins	ISCOMs	BALB/c mice	–	ISCOMs vaccine induces potent serum IgG, and strong IgA responses to RSV locally and remotely in the genital and the intestinal tracts	(65)
	Hepatitis B vaccines	HBsAg	ISCOMs	BALB/c mice	–	ISCOMs prromotes humoral, mucosal, and cellular immune responses	(66)
	OVA model antigen	OVA	IQB-90	Rockfeller mice of the CF-1 breed	–	IQB-90 is a promising alternative to classic ISCOMs as vaccine adjuvants, capable of enhancing humoral and cellular immunity	(67)
	Influenza vaccine	Solubilized envelope glycoproteins	ISCOMs	NMRI mice	PR/8/34(H1N 1)	ISCOMs vaccine induces high levels of antibody and protection against virus challenge.	(74)
	Influenza Vaccines	Split-inactivated influenza vaccine	IQB90	Mice	H1N1 pdm2009	IQB90-adjuvanted influenza vaccine triggs a protective immune response	(75)
	*Eimeria tenella* vaccine	Sporozoite antigens	ISCOMs	Broiler chickens	sporulated oocysts	ISCOMs vaccine reduces the percentage of oocyst shedding and lesion score	(76)
	*Eimeria tenella* vaccine	*E.tenella* total antigens	ISCOMs containing Gg6, Ah6 and Gp7	Broiler chickens	oocysts	ISCOMs vaccine produces higher serum antibodies, increases the weight of broilers, and provides better protection	(77)
	*A. costaricensis* vaccine	A recombinant peptide of PP2A	ISCOMs	C57BL/6 mice	L3 larvae	ISCOMs vaccine partially protects mice against *A. costaricensis* challenge.	(78)
IMX	HTLV-1vaccine	Chimeric peptide	IMX	BALB/c mice	–	IMX vaccine increases antibody titers containing IgG2a, mucosal IgA, IFN-γ and IL-10 cytokines, and decrease the level of TGF-β1, compared to other vaccine formulations.	(68)
	Influenza vaccine	HA	IMX	BALB/c mice sheep	–	IMX vaccine induces serum HAI antibody and mucosal and serum IgA.	(69)
	Influenza vaccine	PR8 antigen	IMX	BALB/c mice	–	PR8- IMX elicits IFN-γ response, IgG1 and IgG2a antibody responses	(70)
	Influenza vaccine	Split MEM vaccine	IMX	BALB/c mice	MEM71 virus	IMX vaccine elicits specific antibody in serum and mucosa when delivered to the entire respiratory tract and protect virus challenge	(79)
	*H. pylori* vaccine	HpaA	ISCOMs/ IMX	Mice	*H. pylori*	HpaA ISCOMs or IMX-vaccine induces protective immunity against *H. pylori* when delivered by the respiratory route	(80)

HSV, herpes simplex virus; gD2, glycoprotein D2; RSV, respiratory syncytial virus; HBsAg, Recombinant hepatitis B surface antigen; OVA ,Ovoalbumin; IQB-90, ISCOMs formulated with the QB-90; *A. costaricensis, Angiostrongylus costaricensis*; HTLV-1, human T-cell lymphotropic virus type 1; HAI, haemagglutination inhibition; PP2A, the serine/threonine phosphatase 2 A. " -" means "not done".

### 3.3 Saponins-based Adjuvant Systems

Since the 1990s, saponins-based adjuvants have moved forward to combine saponins with other adjuvants to provide a synergistic adjuvant effect. GlaxoSmithKline (GSK) biologicals developed several Adjuvant Systems (AS) that incorporate QS-21 with other immunostimulants in different formulations (81). For instance, AS01 is a liposome-based system containing QS-21 and 3-O-desacyl–monophosphoryl lipid A (MPL), while AS02 contains QS-21 and MPL in an oil-in-water emulsion (81). AS05 is a liposome-based system with QS-21, MPL, and alum, while AS15 contains QS-21, MPL, and CpG7909 (82, 83). The U.S. Army has also developed an adjuvant called Army Liposome Formulation containing QS21 (ALFQ) which with different liposome properties to AS01 (84). MPL, a detoxifying lipid from *Salmonella Minnesota* LPS, is a Toll-like receptor 4 (TLR4) agonist and promotes the production of pro-inflammatory cytokines by activating APCs (85, 86). The combination of QS-21 and MPL in AS enhances innate immunity, stimulates antigen-specific T cell responses, and converts mouse antibodies to IgG2c subtype (87, 88). The use of saponins in combination with cholesterol in adjuvant complexes can also reduce hemolytic toxicity. These adjuvant systems have shown good effects in various vaccines against pathogens such as malaria, tuberculosis, SARS-CoV-2, HIV, and *Campylobacter jejuni* (20, 88–93). In particular, AS01 has been licensed for use in the malaria RTS,S/AS01 vaccine and herpes zoster vaccine (Shingrix®) (94). However, there are currently no reports of the use of ASs or ALFQ in nasal vaccines.

AS01, AS05, AS15, and ALFQ are liposome-based adjuvants. Liposomes can protect antigens from degradation and increase antigens absorption across the nasal epithelium, which can prolong the time antigens remain in the respiratory tract (95). They are biocompatible, biodegradable, and safe for nasal vaccine development (95). Intranasal vaccination of influenza vaccine with liposome-based adjuvants has been shown to protect mice from both homologous and heterologous influenza virus challenge (96). Therefore, as liposome-based adjuvants, ASs and ALFQ could be promising options for nasal vaccines development, which needs further investigation.

### 3.4 ISCOMs technology-based Matrix M

Matrix M is the third generation of ISCOMs technology that contains two matrix particles, Matrix-A and Matrix-C, each made from a different, well-characterized saponin fraction from purified Quillaja saponin fractions A and C, along with cholesterol and phospholipids (97, 98). Matrix-C is a highly adjuvant active saponin, while Matrix-A is a weaker but very well-tolerated saponin. The optimal ratio of mixture of Matrix-A and Matrix-C can be explored with co-administering the antigen to achieve the best balance of adjuvant activity and safety (98, 99). Matrix M has been shown to effectively activate and recruit immune cells such as DCs, B cells, and T cells to draining lymph nodes, resulting in strong cellular and humoral immune responses (100, 101). It has been used in clinical trials for vaccines against influenza, malaria, and SARS-CoV-2 (98, 99, 102–104). Interestingly, Matrix-M has mucosal adjuvant properties that enhance mucosal immune responses. Intranasal immunization of Matrix-M (in a ratio of 91:9 of Matrix-A and Matrix–C) adjuvanted a virosomal influenza H5N1 vaccine elicited a significant cross-reactive serum antibody response and protected against a highly pathogenic viral challenge in a mouse model (105). Another study found that intranasal immunization of mice with a Matrix-M-adjuvanted DNA vaccine encoding the P6 outer membrane protein of nontypeable *Haemophilus influenza* resulted in the induction of P6-specific nasal IgA and serum IgG, as well as enhanced bacterial clearance (**Table 3**) (106). These results highlight that Matrix-M adjuvant is a promising mucosal adjuvant for nasal vaccines formulations.

## 4 Understanding the mechanism of action of saponins-based adjuvants in respiratory mucosal responses

**Figure 2:**
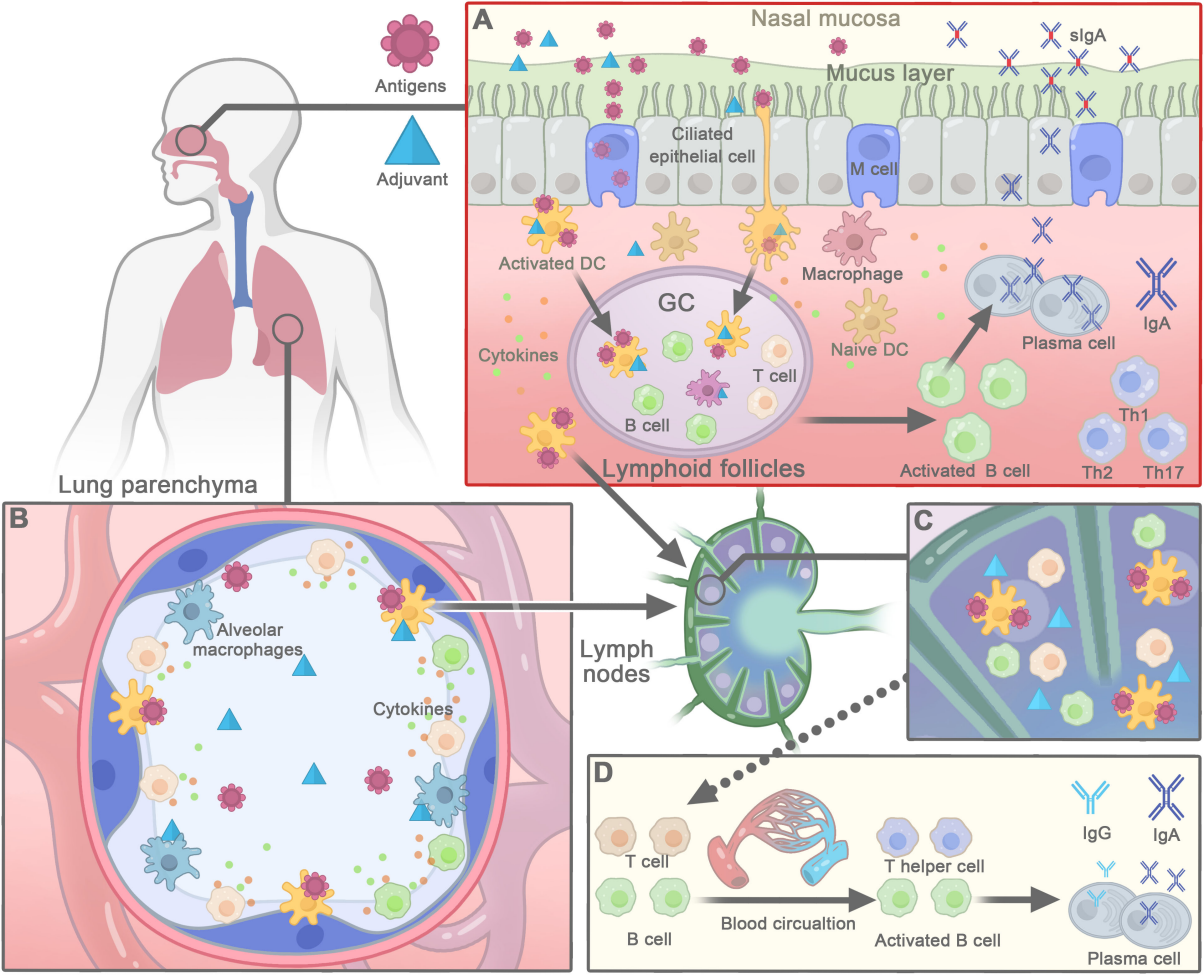
The mechanisms of action of saponins-based adjuvant in nasal vaccines. **(A)** Saponins-based adjuvants promote antigen delivery and uptake by nasal epithelial cells or M cells to the underlying lymphoid follicles. Then dendritic cells (DCs) internalize antigens and adjuvants to be activated and present antigens to stimulate T cells. With the help of activated T cells, the activation of B cells further leads to the formation of the germinal center (GC) in the nasal lymphoid follicles. Afterwards, activated B cells differentiate into plasma cells that secrete IgA, which forms a dimer and is transported back to the effect site of the nasal mucosa, where it provides antigen-specific targeting of respiratory pathogens. Meanwhile, saponins-activated DCs carry antigens to migrate to regional lymph nodes, where they interact with T and B cells to generate antigen-specific T cell and B cells. **(B)** Nasal vaccines, passed through nose to the lung, may also be phagocytized by alveolar macrophages or DCs, and then taken into the interstitium and to hilar lymph nodes. **(C)** In draining lymph node, DCs present antigens to corresponding B- or T-cell to elicit adaptive immune responses. **(D)** Activated B cells and T cells enter the blood circulation to distant systemic or mucosal sites to induce systemic immune responses.

The exact mechanism of action of saponins-based adjuvants after nasal administration is not yet well-understood. However, based on the unique immune structure of the nose and the known mechanism of saponins-based adjuvants (20), the current understanding of how they work as effective adjuvants for nasal vaccine can be reviewed (**Figure 2**).

### 4.1 Delivery of antigens to nasal epithelial cells and APCs

The mucosal immune system is composed of inductive and effector sites. NALT and local and regional cervical draining lymph nodes are induction sites that can trigger both systemic and local mucosal immune responses following nasal vaccination (8, 107). NALT is an organized structure that contains all the immune cells and is covered by a lymphoid epithelium with microfold (M) cells, which are specialized for antigen uptake (8, 107). M cells can transport antigens and adjuvants to APCs, such as DCs, which accumulate immediately below the epithelium and M cells. The APCs phagocytose, process and present antigens to the surrounding naive T cells (108, 109) (**Figure 2A**). Vaccines administered nasally can partially pass into the lungs, where alveolar macrophages take them into the interstitium and to draining lymph nodes to activate DCs (110, 111) (**Figure 2B**). Saponins-based adjuvants in liposome or ISCOMs can deliver antigens to epithelial cells and APCs. The negatively charged IMX nanoparticles have mucoadhesion properties, and the intranasal administration of IMX vaccine has been shown to be retained in the nasal cavity for a longer period of time, allowing antigens to access immune inductive sites in the nasal mucosa and inducing to high mucosal and systemic immune responses (57). Saponins can activate the production of cytokines by resident innate cells, including stromal cells, which then recruit neutrophils, monocyte, and DCs into the respiratory tract to take up more antigens (20).

### 4.2 Induction of proper innate immune response in the local environment.

Proper induction of innate immune response in the respiratory mucosal and draining lymph nodes is crucial for the quality and magnitude of the adaptive responses and vaccine efficacy. Saponins, such as AS01 and QS-21, have been shown to activate and stimulate APCs to release inflammatory cytokines at draining lymph nodes when administrated intramuscularly (88). Saponins can activate innate immune response by binding to the lectin receptors in the innate immune cells, such as DC-SIGN on DCs, and eliciting cytokines and chemokines production in APCs (38, 88, 112) (**Figure 2A**), which leads to a rapid and substantial influx of neutrophils, monocytes, DCs and T-cell populations in the lymph node. AS01 can also enhance the expression of the costimulatory molecules such as CD86 and CD40 on the cell surface of APCs (88). The activated innate immune responses are largely resolved by day 7 (88). These immunostimulant responses could also occur in the respiratory mucosa, NALT, and cervical lymph node, when saponins are delivered intranasally (**Figure 2A**). As intranasal delivery of IMX-adjuvanted human T-cell lymphotropic virus type 1 vaccine was shown to modulate cytokines expression, with increased IFN-γ and IL-10 expression and decreased TGF-β1 levels (68).

### 4.3 Induction of proper adaptive immune responses

The induction of antigen-specific resident memory cells is important for long-term protection against pathogens, which is where mucosal vaccines play a critical role. In NALT or draining lymph node, activated DCs (by saponins and antigens) are capable of activating T cells that differentiate into effector cells and later into memory cells. Activated CD4^+^ T cells, especially follicular helper T cells (Tfh) induce the development of IgA-secreting B cells in NALT or draining lymph nodes (**Figure 2A, C**). AS01 may activate Tfh responses, which are correlated with antigen specific IgG and memory B cells (67, 113–115). The resulting B lymphocytes migrate locally to the lymphatic follicles and proliferate in the germinal center, leading to mucosal and systemic immune responses characterized by the secretion of IgA and IgG, respectively (15). Antigen-specific dimeric immunoglobulin A (dIgA) is transported by epithelial cells through polymeric immunoglobulin receptors (pIgRs) and released as sIgA into the nasal lumen (116, 117) (**Figure 2A**). The antigen and adjuvant-loaded DCs the mature and migrate to the follicular B-cell areas and interfollicular T-cell zone in local draining lymph nodes of nasal tissue or lungs, where they present the antigen to neighboring naive T cells, triggering adaptive immune responses (**Figure 2C**) (106, 118). T cells and B lymphoblasts activated by APCs migrate throughout the body *via* the circulatory system, such as Intranasal immunization of Matrix-M adjuvanted influenza vaccine can activate antigen-specific CD4^+^ and CD8^+^ T cells responses in spleen (105). In addition, immune cells can diffuse through the common mucosal immune system that connects the induction site to the effector sites (**Figure 2D**) (119). Thus, adaptive immune responses are not confined to the site of induction, but also occur at distant mucosal region (63, 119) (**Figure 2D**). Such as, ISCOMs vaccines after nasal administration can induce local antibody secreting cells, CTL, and memory B and T cell responses in lungs (62, 63, 120, 121). The underlying mechanism of saponins to induce mucosal and systemic immune response after intranasal administration requires further study.

### 4.4 Effector sites of respiratory tract

Antigen-specific T cells and IgA^+^ B cells that are induced in NALT and lymph nodes migrate to the mucosa of the respiratory tract through the thoracic duct and circulation. At these effector sites, the IgA^+^ B cells differentiate to IgA^+^ plasma cells to secret sIgA, which is very important in preventing infections by inhibiting the adhesion, invasion, and spread of pathogens to epithelial cells (15, 16, 116) (**Figure 2A**). Intranasal immunizations with ISCOMs vaccines containing different antigens induced not only local IgG and sIgA antibody production in the lungs, but also in distal mucosal system (61, 63–65). The antigen-specific T cells that homing to mucosal region can further differentiate into tissue-resident memory cells that express cell surface markers such as the CD69 and CD103. These memory cells persist in the mucosal tissue for extended periods without entering the circulation and provide a front-line defense against pathogenic invasion by rapidly reactivation in response to antigenic pathogens. Numerous studies have shown that intranasal administration of saponins can induce sIgA production in the respiratory mucosa (**Tables 1**-**3**) (**Figure 2A**) (44, 50, 53, 61, 64, 68). Further research is needed to understand the regulation of tissue-resident memory T cell and B cell responses triggered by nasal administration of saponins.

**Table 3:** Matrix-M used as adjuvants in nasal vaccines.

Vaccine	Antigen	Adjuvant	Animal model	Pathogen Challenge	Main findings	Reference
**Influenza vaccine**	Virosomal influenza A H5N1 vaccine	Matrix-M	BALB/c mice	A/Vietnam/1194/2004 (H5N1) virus.	Matrix-M adjuvanted virosomal vaccine induces influenza specific CD4^+^ and CD8^+^ T cells and protects against virus challenges	(105)
**NTHi vaccine**	DNA plasmid encoding the P6 outer membrane protein	Matrix-M	BALB/c mice	NTHi (strain 76)	Matrix-M DNA vaccine induces P6-specific IgA, serum IgG and IgA-producing cells in the nasal passages and induces P6-specific Th1, Th2 and Th17 responses in NALT. Matrix-M DNA vaccine enhances bacterial clearance	(106)

NTHi, nontypeable Haemophilus influenza (NTHi); NALT, Nasal-associated lymphoid tissue.

## 5 The potential disadvantages of saponins-based adjuvants for nasal vaccine

### 5.1 The safety of saponins-based adjuvants for nasal vaccine

Ensuring the safety of the effective adjuvants is the first priority in vaccine development, particularly for those based on saponins used in nasal vaccine, where their safety profile in humans is not yet fully established. Saponins are amphiphilic compounds containing both hydrophobic and hydrophilic regions and their hydrophobic regions can interact with the cell membranes of red blood cells and disrupt the membrane integrity, leading to hemolysis. To mitigate the toxicity, saponins can be formulated with other components, such as cholesterol used in AS01 and ISCOMs, which can interact with the hydrophobic region of saponins to prevent their interaction with cell membranes (122). The adjuvant effects of saponins are dependent on their immunostimulatory effect, which can stimulate transient inflammation at the injection site. The effects usually are mild and transient, returning to normal within several days after administration. However, high dose of adjuvants may cause strong inflammation or cell death. Although saponins and saponin-based adjuvants are generally considered safe for parenteral immunization (93, 102, 106 (90, 99, 102, 103, 106, 123), they have been associated with some adverse effects, including local pain, redness, swelling, and fever, especially at the injection site (124, 125). So, intranasal administration of saponin-based adjuvants may induce inflammation and cellular damage in respiratory tract and lungs at high doses, which is a safety concern that requires careful evaluation in the development of nasal vaccines (50). Another important safety concern for nasal adjuvants is their potential side-effects on the central nervous system, since they may be transported from the olfactory epithelium to central nervous system (126, 127). Thus, evaluating the local and systemic toxicities of nasal vaccines containing saponin-derived adjuvants is crucial, which should be carefully evaluated for a good balance of efficacy and side effects in pre-clinical and clinical studies.

### 5.2 Challenges in developing saponins-based adjuvants for nasal vaccine

Successful development of saponin-based adjuvants for nasal vaccines requires consideration of the unique physiological, chemical, and immunological properties of the nasal cavity. The physiobiological barrier system, including the mucus and mucociliary movement, may hinder antigens and adjuvants absorption (14). Nasal mucus containing proteases and aminopeptidases may degrade the vaccine components (14). However, saponins-based adjuvant such as ASs or ISCOMs, which use liposome as a delivery system, may overcome these barriers and transport antigens and adjuvants to epithelial cells and APCs, which require further evaluation. Additionally, administering vaccines through the nasal cavity may result in the passage of vaccines into the lungs or oral cavity, making it difficult to determine the exact amount of antigens or adjuvants that reach the immune system. To address this issue, metered-dose nasal spray devices can be used to control the amount of solution delivered per spray, allowing for the evaluation of efficacy and side effects to determine the optimal spray dose. In conclusion, overcoming these obstacles is critical when developing saponins-based adjuvant for nasal vaccines.

## 6 Future perspective of saponin-based adjuvants

### 6.1 Combination of saponin-based adjuvants with other adjuvants

Different adjuvants have unique immunomodulatory effects and the protective immune responses for different pathogens can vary. To achieve enhanced and broad protective immune responses for co-administered antigens, a combination of saponins with different mucosal adjuvants or delivery systems can be used, potentially resulting in additive enhancing effects on mucosal immune responses

AS01 is a successful example of combining two adjuvants, MPL and QS-21, in one formulation. Other attempt to combine different adjuvants includes intranasal administration of the ginseng stem-leaf saponins (GSLS) in combination with selenium (GSLS-Se). This combination enhanced the adjuvant effect on live vaccines for Newcastle disease virus and infectious bronchitis virus in chickens, promoting significantly higher antigen-specific antibody responses, increased lymphocyte proliferation and production of IFN-γ and IL-4 compared to GSLS alone. The increased antibody was able to neutralize corresponding viruses (128). Antigen-specific sIgA and the numbers of IgG^+^, IgA^+^, IgM^+^ plasma cells were significantly higher in GSLS-Se group than the control in the Harderian gland (129). Another example is the combination of cyclic guanosine monophosphate-adenosine monophosphate (cGAMP) and saponins, which improved protective response to influenza. Saponins can increase the permeability of cell membrane, allowing more cGAMP to enter the cells and increasing the utilization rate of saponins, leading to stronger immune effects (130, 131). Furthermore, nasal delivery systems such as microspheres or nanospheres made of chitosan, PLGA (poly[D,L-lactic-co-glycolic acid]), alginate, or cross-linked dextran have been employed to encapsulate saponins for nasal administration (132–135). These studies provide compelling evidence for the potential of developing mucosal adjuvants through combinations of saponins and other adjuvants.

ISCOMs and IMX complexes are also a promising option for the development of combined adjuvant carriers with other adjuvants to increase adjuvant activity. Combination of a mucosal adjuvant cholera toxin B (CTB) with ISCOMs has shown increased adjuvant effects. Intranasal immunization of synthetic peptide polymerized with the CTB in ISCOMs showed increased protection against polymerized synthetic peptide in ISCOMs (78). In addition, the cholera toxin subunit A1 (CTA1) fused to a dimer of the Ig-binding D-region of *Staphylococcus aureus *protein A (CTA1-DD) has been incorporated into ISCOMs to create CTA1-DD/ISCOMs adjuvant. This adjuvant significantly augments the immunogenicity of the antigen, as demonstrated by increased levels of specific serum antibodies, balanced Th1 and Th2 priming, and strong activation of DCs (136–138). Moreover, intranasal vaccination with CTA1-DD/ISCOMs adjuvanted antigens Ag85B-ESAT-6 from *M. tuberculosis* significantly reduced the *M. tuberculosis* burden in the lungs compared to control animals (139). However, combination of adjuvants does not always show enhanced effects. For instance, the combination of chitosan with IMX nanoparticles in a vaccine using the PR8 antigen induced a weaker immune response compared to IMX nanoparticles alone after intranasal administration (70).

### 6.2 Combination of different immunization routes of adjuvants

Combining different routes of adjuvant delivery through prime-boost strategies has the potential to enhance both mucosal and systemic immune responses. For example, a systemic prime-intranasal boost strategy with an influenza vaccine adjuvanted with the liposomal dual TLR4/7 adjuvant has been shown to enhance both systemic and local/mucosal immunity. This regimen results in the secretion of antigen-specific sIgA and development of tissue-resident memory T cells in the respiratory tracts, as well as cross-reactive sIgA to multiple influenza virus strains (140). Similarly, this prime-boost strategy has been successful in preventing SARS-CoV-2 transmission and disease development through vaccination (141). Therefore, combining different routes of adjuvant delivery can provide comprehensive and effective immune responses at both the systemic and mucosal levels. Such strategies can also be applied to the study of saponin-based adjuvants in nasal vaccines. However, the optimal combination of administration routes may vary depending on the specific vaccine and pathogen being targeted, and thus requires thoroughly experimentation.

### 6.3 Understanding the mechanism of adjuvants through new technologies

Achieving effective vaccine-induced immune responses requires a deeper understanding of the mode of action of saponin-based adjuvants, which can expedite the development of novel vaccine strategies. Collaborative research efforts between various disciplines, including chemistry, biochemistry, molecular biology, immunology, material science, and artificial intelligence, are crucial to achieve this goal. By using of multiomics technology such as transcriptomics, proteomics, and metabolomics at bulk and single-cell levels, researchers can uncover the function and mechanism of saponins-based adjuvants. Machine learning algorithms have also been applied to identify immune signatures associated with adjuvant formulations like AS01B, AS02A, AS03, CpG, and MF59 (142–144). Combining machine learning with in-depth profiling of vaccine-induced immune signatures including cytokine, cellular, and antibody responses can lead to identify adjuvant-specific immune response characteristics that can predict the efficacy and safety of the adjuvants in human (142–145). Moreover, immune response patterns in mice may not be predictive of responses in human (146). Therefore, organoids derived from lymphoid tissues, such as tonsils, will proved a powerful platform for studying key immune mechanisms related to human and enable rapid preclinical research on saponins-based adjuvants (146).

## 7 Conclusion

Saponin-based adjuvants have been demonstrated to have minimal side effects and are relatively non-toxic. Their administration *via* the nasal route has been shown to enhance the immune response, making them an appealing option for the development of nasal vaccines. This review aims to highlight the potential of saponins-based adjuvants for respiratory mucosal vaccines, offer further adjuvants candidates for the purpose of rational delivery system design, and ultimately drive the progress in the field of nasal vaccine development. We hope that this review will provide valuable insights and stimulate further research in this field.

## Author contributions

YS conceived the ideas and finalized the review. KC drafted the manuscript. NW, XZ, MW, YL participated for collecting the materials. All authors contributed to the article and approved the submitted version.
